# ProDaMa: an open source Python library to generate protein structure datasets

**DOI:** 10.1186/1756-0500-2-202

**Published:** 2009-10-02

**Authors:** Giuliano Armano, Andrea Manconi

**Affiliations:** 1 Department of Electrical and Electronic Engineering, University of Cagliari, P.zza D'Armi, 09123 Cagliari, Italy

## Abstract

**Background:**

The huge difference between the number of known sequences and known tertiary structures has justified the use of automated methods for protein analysis. Although a general methodology to solve these problems has not been yet devised, researchers are engaged in developing more accurate techniques and algorithms whose training plays a relevant role in determining their performance. From this perspective, particular importance is given to the training data used in experiments, and researchers are often engaged in the generation of specialized datasets that meet their requirements.

**Findings:**

To facilitate the task of generating specialized datasets we devised and implemented ProDaMa, an open source Python library than provides classes for retrieving, organizing, updating, analyzing, and filtering protein data.

**Conclusion:**

ProDaMa has been used to generate specialized datasets useful for secondary structure prediction and to develop a collaborative web application aimed at generating and sharing protein structure datasets. The library, the related database, and the documentation are freely available at the URL .

## Introduction

Notwithstanding the growth in experimental data on protein structures, the difference between the number of known sequences and known tertiary structures is still very large and growing steadily. This discrepancy has justified the use of automated methods of protein sequence analysis that has led to the development of various predictors, such as systems to predict protein secondary structure (e.g. [1,2]), transmembrane regions (e.g. [3]) or beta-turns (e.g. [4,5]). Despite the increase in accuracy, a general methodology to solve these problems has not yet been devised. The accuracy of these systems is also related to the examples used for training. Different protein datasets have been proposed in the literature to investigate specific problems. However, these datasets may not be in accordance with the needs of researchers, or may not fit the specific nature of the problem. Owing to these limitations, researchers must often generate themselves datasets able to satisfy their needs. To this end, they use specialized databases, tools to browse them, and tools to analyze the data stored therein. To generate a dataset, a researcher must interact with these tools separately and overcome the limitations associated with the migration of data from one tool to another, and with the methods available for managing the data. From this perspective, the Biopython [6] library takes an important role. To help reserchers in the task of managing bioinformatics data, Biopython provides a set of tools mainly aimed at *i) *parsing bioinformatics files into Python data structures, *ii) *dealing with a set of popular on-line bioinformatics resources, and *iii) *interfacing to common bioinformatics programs. In order to generate protein structure datasets, major benefits can be obtained with a set of specialized tools for automatically retrieving and organizing relevant protein data, as well as analyzing and selecting them according to specific constraints that apply to their sequence and/or structure. To facilitate this task we developed ProDaMa (**Pro**tein **Da**tasets **Ma**nagement), an open source Python library aimed at helping researchers in the task of generating protein structure datasets able to meet their requirements. ProDaMa is designed for: *i) *retrieving protein data from several remote sources, *ii) *organizing and storing them in a local database, and *iii) *analyzing and filtering them to generate specialized datasets according to user-defined criteria.

## Retrieving Protein Data

ProDaMa allows one to retrieve data from a set of selected remote bioinformatics sources. In particular: *i) *proteins from the Protein Data Bank (PDB) [7], *ii) *information about protein structure classification from CATH [8] and SCOP [9], *iii) *other protein data from the PDBFINDER database [10], and *iv) *information about membrane protein topologies from the MPTopo database [11].

## Organizing and Updating Data

To store and organize data, a MySQL local database has been devised and implemented. For each protein the database stores: its identifier, its primary and secondary structure, data associated with the CATH classification, data associated with the SCOP classification, and information retrieved from the PDBFINDER database. For membrane proteins, the database also stores information about their topology and their membership of the generated datasets. To take into account changes in PDB files, ProDaMa provides functionalities to update the local database. Updating is performed in three steps: *i) *look for obsolete proteins in the PDB and remove the corresponding data from the local database, *ii) *look for new proteins in the PDB, and *iii) *retrieve and store information associated with these proteins.

The database has been pre-loaded with proteins from the PDB, as well as with a number of commonly used biological datasets. In particular RS126 [12], PDBSELECT25 [13], and the clusters of structures based on 50%, 70%, 90% and 95% sequence identity from PDB. The datasets of sequence structures used by WHAT IF [14], based on sequence identity, resolution and R-factor, have also been pre-loaded.

## Analysing and Filtering Data

With ProDaMa new datasets can be generated and made available starting from the content of the local database or from any previously-generated dataset. In both cases the information source flows through a pipeline of methods/operators, with the obvious constraint that their input/output compatibility along the pipeline must be ensured. Four groups of methods/operators are available off-the-shelf: *i) *search methods, *ii) *filter operators, *iii) *set operators, and *iv) *encoding methods. *Search methods *are typically applied to the local database, to select proteins that satisfy homology and/or similarity constraints. In particular, FASTA [15] and PSI-BLAST [16] algorithms, useful to perform search by sequence similarity, are available in the form of web service calls, while PISCES [17], aimed at performing searches by sequence identity, has been integrated in ProDaMa (PISCES is used for culling sets of protein sequences from the PDB or from an existing dataset, according to sequence identity and structural criteria). Methods for CATH and SCOP protein similarity searching, as well as for transmembrane protein topology search, are also provided. Furthermore, proteins can be selected by imposing constraints on their quality -i.e., on the experimental method that has been used, on the X-ray resolution, as well as on their R-factor and free R-factor. *Filter operators *are aimed at selecting relevant proteins according to a unary predicate (e.g., from the input dataset select only single-chain proteins) or according to a binary predicate (e.g., from the input dataset select proteins with a percent of identity ≤ 25%). In the latter case, a protein culling tool is required. Currently, only PISCES is made available for this purpose. *Set operators *currently supported are the classical union, intersection, and difference. *Encoding methods *are aimed at mapping the primary structure of a protein -given in terms of the IUPAC standard encoding- to other relevant alphabets (chemical, physical, and hydrophobic alphabets are currently available off-the-shelf). Alternatively, the primary structure can be mapped to an amino acid index [18]. Any generated dataset can be (and typically is) stored in the local database ready to be used or updated according to the user's needs. Of course, any such dataset can become a source for further pipelines of methods/operators devised to generate new datasets. Some examples follow, aimed at demonstrating the potential and the ease of use of ProDaMa in the task of analyzing and filtering data.

*Example 1 - *Shows how to manage a dataset according to the protein quality parameters, and to the structure composition. Here the structures in the dataset PDBSELECT25 are restricted to those solved by X-ray crystallography, with a maximum R-factor of 0.2, and a minimum helical content of 10% using specialized search methods.

# Get the selected dataset

dataset = Dataset('PDB-Select25')

# Look for proteins according to the constraints on the their quality

ids = dataset.lookForProteinQuality(exp_method = 'X', MAX_rfactor = 0.2)

# Look for proteins according to the constraint on the structure composition

ids = Dataset(ids).lookForStructureComposition(label = 'H', MIN = 0.1)

*Example 2 - *Shows how to manage a dataset according to the classification of protein domain structures. A dataset, obtained by removing multichain proteins from those that meet a given constraint on their CATH classification, is furthermore restricted using a filter operator aimed at reducing sequence redundancy. Chains are filtered by disregarding sequences with identity above 30% and length lower than 80 residues.

# Look for proteins in the database that meet a specific constraint at the

# CATH class and architecture classification level

ids = Dataset().lookForCATHClassification(class = 'Alpha Beta', architecture = 'Alpha-Beta Complex')

# Look for single-chain proteins in the dataset

ids = Dataset(ids).selectChain(mode = 'single')

# Cull a set of protein sequences from the dataset according to sequence identity

# and structural criteria

ids = Dataset(ids).sequencesCull(MAX_percentage_identity = 30, MIN_chain_length = 80)

*Example 3 - *Shows how to generate a non-redundant dataset of transmembrane proteins that meet a given constraint on their topology, and on their transmembrane segment length. The resulting dataset is intended to be used for comparative assessment of transmembrane protein predictors.

# Let "datasets" be a list of the datasets used to train a set of predictors

# to be assessed

# Look for alpha-helical transmembrane proteins

ids = Dataset().lookForTMTopology(topology = 'alpha helical')

# Select only proteins characterized by transmembrane segment length within

# a given range (9-18) ids = Dataset(ids).lookForTMSegments(MIN = 9, MAX = 18)

# Select proteins with a maximum of 25% pairwise sequence identity

ids = Dataset(ids).sequencesCull(MAX_percentage_identity = 25)

# Remove from the dataset all proteins used to train predictors subject to

# comparative assessment

dataset = Dataset(ids)

i = list()

for d in datasets: i.append(dataset.intersection(d))

dataset = dataset.difference(i)

*Example 4 - *Shows how to perform statistical analysis on a dataset.

# Let "myDataset" be the name of a dataset previously generated and stored into

# the local database

# Get the dataset

dataset = Dataset('myDataset')

# The size of the dataset (nb of chains)

size = len(dataset.getIds())

# The total number of aminoacids in the dataset

nb_aa = dataset.length()

# The average length of a sequence in the dataset

average_length = dataset.averageLength()

# Analyze the sequence composition of the proteins in the dataset according

# to the aminoacid, chemical, functional, and hydrophobic standard alphabets

aa_statistics = dataset.getSequenceStatistics()

che_statistics = dataset.getSequenceStatistics(alphabet = 'che')

fun_statistics = dataset.getSequenceStatistics(alphabet = 'fun')

hyd_statistics = dataset.getSequenceStatistics(alphabet = 'hyd')

## Conclusion

Protein sequence analysis is an important research area in bioinformatics owing to the huge difference between the number of known sequences and known tertiary structures which has led to the development of automated methods of analysis. The choice of the training dataset strongly affects the accuracy of the system being implemented. In the literature, different protein structure datasets are proposed, but they do not always meet the requirements of researchers. To help them construct specialized datasets we developed ProDaMa, an open-source Python library that permits one to retrieve protein data from a number of remote sources, to organize and store these data in a local database, and to construct specialized datasets by analyzing and selecting those proteins that fulfill user-defined criteria. ProDaMa has been used to develop ProDaMa-C [19], a collaborative web application aimed at helping researchers to generate and share protein structure datasets. It is worth noting that the current release of ProDaMa-C embeds only part of the ProDaMa functionality. In future work, we plan to embed all ProDaMa functionality in ProDaMa-C.

## Availability and requirements

• **Project name**: Pro.Da.Ma.

• **Project home page**: 

• **Operating System**: Linux

• **Programming language**: Python 2.6

• **Other Requirements**: MySQL 5.0

• **Licence**: GNU GPL

## Competing interests

The authors declare that they have no competing interests.

## Authors' contributions

Both authors contribute equally to devising the library.
